# Pre-operative risk scores for the prediction of outcome in elderly people who require emergency surgery

**DOI:** 10.1186/1749-7922-2-16

**Published:** 2007-06-05

**Authors:** Thomas E Rix, Tom Bates

**Affiliations:** 1 Department of General Surgery, Eastbourne District General Hospital, Eastbourne, East Sussex, BN21 2UD, UK; 2 Kent Institute of Medicine and Health Sciences, University of Kent, Canterbury, Kent, CT2 7PD, UK

## Abstract

**Background:**

The decision on whether to operate on a sick elderly person with an intra-abdominal emergency is one of the most difficult in general surgery. A predictive risk-score would be of great value in this situation.

**Methods:**

A Medline search was performed to identify those predictive risk-scores relevant to sick elderly patients in whom emergency surgery might be life-saving.

**Results:**

Many of the risk scores for surgical patients include the operative findings or require tests which are not available in the acute situation. Most of the relevant studies include younger patients and elective surgery. The Glasgow Aneurysm Score and Hardman Index are specific to ruptured aortic aneurysm while the Boey Score and the Hacetteppe Score are specific to perforated peptic ulcer. The Reiss Index and Fitness Score can be used pre-operatively if the elements of the score can be completed in time. The ASA score, which includes a significant element of subjective clinical judgement, can be augmented with factors such as age and urgency of surgery but no test has a negative predictive value sufficient to recommend against surgical intervention without clinical input.

**Conclusion:**

Risk scores may be helpful in sick elderly patients needing emergency abdominal surgery but an experienced clinical opinion is still essential.

## Background

When an elderly person presents to hospital requiring an emergency operation, it is the surgeon and anaesthetist's duty to assess the risks of anaesthesia and surgery by establishing the patient's pre-morbid condition, quality of life (QOL) and prognosis and to weigh this with the likelihood that a surgical operation will be successful. Surgery may not be advised if the chance of success is slight and the risk of causing a fatal outcome is considered greater by operating. In order to make these difficult decisions more objective a number of scoring systems have been constructed. The most commonly used is the American Society of Anaesthesiologists (ASA) score which includes a category, ASA V, for patients who are not expected to survive whether or not surgery is performed [1]. This group of patients has also been categorised as "non-viable", a term favoured by Seymour and Pringle [2] who advocate leaving out this group of dying patients when auditing a surgical unit's mortality figures.

Ideally, surgeons would have a reliable, easy-to-calculate scoring system to apply to all elderly people presenting for emergency surgery. An accurate prediction of outcome could then be made while assessing a patient's fitness for surgery, allowing the surgical team to present a more informed choice to the patient on whether surgery or supportive care is the optimal management. By providing a percentage risk of mortality, of morbidity, or of post-operative reduction in QOL, such a score would also be helpful when communicating with the most unfit patients and their relatives, why surgery is not advised.

Although easy to use, the ASA score has not fulfilled all of these ideals and many other scores have been developed [3-8]. Some of these are only useful in elective surgery since they require tests that would not be practical in the assessment of a patient for an emergency laparotomy.

Most surgical risk scores have been created from large operative databases, using statistical analyses to determine which variables are most-strongly associated with outcome. These scores, of which the most-commonly used here in the UK is the Physiological and Operative Severity Score for the enUmeration of Mortality (POSSUM) [6] or one of its modifications [7], usually require intra-operative information, and so are not useful in the pre-operative assessment phase of management. Indeed, this was not their intended use. They were designed for comparative surgical audit, to discount the effect of case-mix on outcome. The Acute Physiology and Chronic Health Evaluation (APACHE) score [8] or the later version APACHE II, is commonly used to assess surgical patients on the Intensive Care Unit (ICU) where it was designed to predict outcome, but it has seldom been used in pre-operatively assessment [9].

The purpose of this review is to establish the current status of pre-operative scoring systems in emergency surgery, focussing on decision-making in elderly people. Is there a reliable and accurate scoring system to help decide if an elderly person being considered for an emergency laparotomy will be made better or worse by surgery?

## Methods

A search of the literature from 1977 – 2007 was performed using Medline. The search terms used included: elderly, aged, pre-operative assessment, emergency surgery and scoring. Secondary references were obtained from key articles. Although articles relevant to other surgical specialties were assessed, the focus was on abdominal and vascular emergencies.

## Results

Most of the literature on surgical scoring systems concerns both elective and emergency surgery, and although there is a great deal of literature on risk-management in elderly people, there is relatively little which is specific to the subject of this review.

Many publications describe the use of post-operative scoring systems such as POSSUM to compare predicted and observed outcomes, but this review is focussed on practical scores which can be calculated pre-operatively in an elderly surgical patient with peritonitis. Systems are included if they apply to all age groups but will be of most relevance in the elderly, such as ruptured abdominal aortic aneurysm (AAA) scores. Likewise, scoring systems which were validated on a mixture of acute and elective cases have been included if they provide a useful outcome prediction in emergency surgery.

### Outcome measures

Operative mortality is the most commonly-used marker of outcome after surgery and most scoring systems aim to predict post-operative death. Patients may well consider other outcome measures, such as the length of recovery time and QOL as more important. Some scoring systems aim to predict the risk of surgical complications, but none were found that make QOL predictions after emergency surgery. This compares unfavourably with the situation in elderly care medicine where scoring systems such as the Sickness Impact Profile do exist for predicting the effect of interventions on QOL after discharge from hospital [10]. Table 1 lists the scoring systems identified in this review, classified by outcome measure.

**Table 1:** Surgical risk scores classified by outcome measure and need for intra-operative information

	**Scores predicting mortality**	**Scores predicting morbidity**
**Scores not requiring operative information**	ASA^1^	ASA
	APACHE-II^8^	APACHE-II
	Donati Score^16^	Goldman Cardiac Risk Index^3^
	Hardman Index^38^	Veltkamp Score^44^
	Glasgow Aneurysm Score^12^	VA Respiratory Failure Score^45^
	Sickness Assessment^14^	VA Pneumonia Prediction Index^46^
	Boey Score^32^	
	Hacetteppe Score^34^	
	Physiological POSSUM^35^	
**Scores requiring operative information**	Mannheim Peritonitis Index^28^	POSSUM, P-POSSUM
	Reiss Index^11^	
	Fitness Score^13^	
	POSSUM^6^, P-POSSUM^7^	
	Cleveland Colorectal Model^43^	
	Surgical Risk Scale^47^	

### Creation of scoring systems

It has proven difficult to design scoring systems that are both accurate and easy to use. Scores derived by multivariate analysis of a large cohort of patients, such as the Reiss Index [11] usually require several pre-operative data to be collected and diagnostic information that may not be available until surgery is undertaken. Scores that are simpler to apply in practice have either been derived from multi-variate analysis, such as the Glasgow Aneurysm Score [12], or from an arbitrary selection of likely risk factors that the authors have weighted without statistical analysis, such as the Fitness Score [13].

## Pre-operative scoring systems

### 1. Age

Using the patient's age alone as a 'score' to determine whether or not they will be fit to survive surgery has been shown to be invalid. There is now a large amount of evidence that demonstrates that although older patients do worse after emergency surgery, with mortality rates in over-74 year-olds double that of 65–74 year-old in one UK study [14], it is because they have more co-morbidities than younger patients. In multi-variate analyses, age on its own has been shown to be a poor predictor of mortality, morbidity or length of stay in hospital [15]. APACHE-II scores appear to predict outcome equally well when the age points are omitted [9]. A fit elderly person should not be denied an emergency operation because of their age alone.

### 2. ASA score

Although the ASA classification of fitness for surgery was not devised as a risk prediction score, it has been used in this way, both on its own, and in conjunction with other patient or operative variables such as age [16-18], sex [18], urgency of surgery [16] or APACHE-II score [9]. Several studies have described the association between ASA score and observed post-operative mortality in elderly patients undergoing emergency gastrointestinal surgery (Table 2a/2b).

**Table 2a:** Observed mortality in emergency surgery in the elderly by ASA grade

**Study**	**n**	**Age (≥)**	**ASA I**	**ASA II**	**ASA III**	**ASA IV**	**ASA V**
Arenal^15^	710	70	6%		19%	38%	89%
Barlow^14^	204	64	0%	9%		50%	75%
Cook^18^	107	65	0%	17%	25%	77%	91%
Akoh^19^	83	80	-	13%	25%	75%	-
Makela^20^	71*	70	0%	0%	9%	29%	100%
Cook^50^	49	75	0%	0%	10%	63%	100%

Combined boxes when study has combined two ASA classes for analysis; -indicates no patients with this ASA score in study; * all 71 patients had perforated diverticular disease

**Table 2b:** Summary of the 6 studies observing mortality after emergency surgery in the elderly

	**ASA I**	**ASA II**	**ASA III**	**ASA IV**	**ASA V**
Total deaths	31		62	143	41
Total cases	511		338	329	46
Mortality	**6%**		**18%**	**44%**	**89%**

The rate of post-operative morbidity in each ASA class has also been observed. Akoh *et al *found morbidity rates after emergency laparotomy of 40%, 63% and 100% in ASA 2, 3 and 4 patients respectively [19]. 64% of all ASA 2–5 patients in the study by Barlow *et al *developed post-operative complications [14].

In uni-variate and multi-variate analyses of emergency surgical patients and mortality, ASA has consistently been shown to be a good predictor of death post-operatively [15,20,21]. This is in spite of its subjective nature and the inter-observer variation in measuring ASA [22].

A recent Italian study has used ASA in conjunction with urgency of surgery (elective vs. urgent/emergency) and patient age (< 50; 50–69; > 70years) to develop a model for predicting mortality after surgery that could be used pre-operatively [16]. This model was developed on data from 1936 patients and validated on a further 1849 patients, although 95% of these were elective cases. The prediction of mortality using this system is shown in Table 3. The definition of major surgery used in the article would include all emergency laparotomies involving a bowel resection or vascular procedure. By Receiver Operator Characteristic (ROC) curve analysis this score was shown to be a slightly more accurate predictor than ASA alone. (Area under the ROC curve equals the probability of concordance between the predicted and observed mortality, from 0.5 representing chance performance to 1 for perfect performance).

**Table 3:** Predicted risk of mortality after major surgery performed as urgent/emergency. (Adapted from Donati et al^16^)

**ASA Class**	**Age 50–69**	**Age ≥ 70**
I	2%	0%
II	8.2%	12.9%
III	21%	30.6%
IV	44.3%	56.8%

ASA has been used for many years, and remains the only score routinely used in most surgical emergency cases. It was not designed to predict mortality but it has been shown to give a good estimate of mortality risk with the great advantage of being simple to score. It is subjective however, and may be applied inconsistently by different anaesthetists.

### 3. APACHE-II

The APACHE-II score has been studied for its pre-operative predictive value as well as for the intended purpose of predicting postoperative mortality on ICU. It is relatively easy to calculate in the emergency assessment setting, but the acquisition of 12 physiological variables will always make it less readily measured than ASA.

Goffi *et al *measured APACHE II and ASA scores pre-operatively in 187 general surgical patients, of which 49 were emergency cases, and compared their accuracy at predicting mortality and morbidity in the first 30 post-operative days [9]. APACHE II gave a better prediction of outcome than ASA when assessed with ROC curves. The area under the ROC curve for both elective and emergency cases was 0.894 for APACHE II compared to 0.777 for ASA (p < 0.001). There was no significant difference between prediction in emergency or elective cases, nor between the prediction pre or post-operatively. It was concluded that APACHE II may be cautiously used pre-operatively, but not as a substitute for good clinical judgement.

### 4. Sickness assessment

Kennedy *et al *prospectively analysed emergency surgical admissions in elderly patients [23]. 498 consecutive surgical admissions were studied, of patients aged 65 and above. Their simple scoring system, the Sickness Assessment (SA), used three variables: hypotension; severe chronic disease and whether or not the patient was independent and self-caring. These conditions were clearly defined. In the group of patients with a SA score of zero, there were no deaths. Mortality in patients with one, two and three parameters present was 52%, 60% and 100% respectively. Three quarters of all deaths were predicted. Hypotension (Systolic BP < 100 mmHg on admission) carried the highest predictive power; mortality was 77% in patients undergoing laparotomy who were hypotensive on admission (sensitivity 61%). Laparotomy in the presence of a positive SA was associated with a 57% mortality compared to 15% in those with a zero SA (p < 0.001). The APACHE II system was not found to have superior predictive power, however a score of only 12 was used as a cut off. Increasing the cut-off increased specificity but reduced sensitivity. No ROC curve analysis was performed on these data.

### 5. Fitness score

A study by Playforth *et al *in 1987 describes a scoring system applied to 1517 consecutive patients of all ages undergoing major abdominal surgery [13]. Emergency operations accounted for 50% of this cohort and 46% of these patients were over 70 years old. The 26 risk factors were chosen by the authors and weighted arbitrarily from 1 – 4 with, for example, Haemoglobin < 10 g/dL scoring 1 and age > 80 years scoring 4. This method calls into doubt the likely validity of this scoring system. Patients scoring less than 6 were found to have a mortality rate of 0.7%; compared to 38% with a score of 6 or more. The sensitivity of the Fitness Score was 96% and specificity 81% when 6 was used as the cut-off between low and high-risk.

In addition to the difficulty of scoring 26 variables pre-operatively, some, such as the presence of perforation or obstruction and diagnosis of cancer, may not be available before surgery

### 6. Reiss index

Reiss *et al *have produced a scoring system for predicting mortality in the elderly [11]. Their study is exclusively focussed on laparotomies in old people, but not on emergencies alone. Using 36 variables, acquired from 1200 patients undergoing laparotomy, they identified the five most significant factors by multi-factorial stepwise regression analysis. These were: age; urgency of surgery; ASA; presence of malignancy and diagnosis. The score was validated pre-operatively on another 200 patients.

An emergency laparotomy where the diagnosis was unknown could not be scored with this system, which has been shown to be inferior to the ASA classification in predicting postoperative morbidity and mortality. In an Italian study, 125 consecutive patients, aged older 70 years, undergoing surgical treatment, were investigated [24]. The patients were grouped according to the ASA score and Reiss Index. Post-operative morbidity and mortality rates were calculated. Both indices were good predictors of postoperative prognosis but the sensitivity of the ASA score was better.

### 7. Sepsis scores

As well as the APACHE score, several other scoring systems have been developed for intra-abdominal sepsis. These scores include the Simplified Acute Physiology Score (SAPS) [25], Sepsis Score [26], Multiple Organ Failure Score [27] and Mannheim Peritonitis Index (MPI) [28]. In comparative studies the APACHE II and MPI appear to offer the best prediction of outcome. As in most studies of scoring systems, Bosscha *et al *[29] point out that the MPI, though the best of these scores at predicting outcome, could not be used on individual patients as a decision-making tool due to its low specificity. Qureshi *et al *also found a high false positive rate (72%) using MPI alone [30]. Combining the MPI and APACHE II improves specificity [29] but these scores are most useful in auditing outcome for a group of surgical ICU patients [31].

### 8. Peptic ulcer scoring systems

#### a) Boey score

Having determined a group of risk factors for mortality in perforated peptic ulcer, Boey *et al *in 1987 validated these factors in a second cohort of 259 consecutive patients with perforated peptic ulcers undergoing emergency laparotomy [32]. Mean age was only 51.3 years. They identified three risk factors and a risk of mortality associated with the number present in each case, as shown in Table 4. There were no false-negative errors – all 16 patients who died post-operatively were identified with this system. However a false-positive rate of 53% means that if the decision not to operate had been based on a positive mortality prediction with this score, 28 life-saving operations would have been declined.

**Table 4:** Mortality risk from perforated peptic ulcer according to Boey et al ^32^, and Irvin^33^

**Risk Factors**	**No. of Risk Factors**	**Risk of Mortality**	
		**Boey**	**Irvin**
	
	0	0	11%
Preoperative BP < 100 mmHg	1	10%	27%
Delayed presentation > 24 h	2	45.5%	55%
Major medical illness present	3	100%	100%

Irvin [33] attempted to validate the Boey Score on a cohort of 265 consecutive patients who had operations for perforated peptic ulcer. 176 of these were 70 years or above, of which two-thirds were female. All 5 patients with three Boey Score risk factors died. At a cut-off of two risk factors the accuracy was less good, with 13 patients surviving from 29 (false positive rate 45%) Mortality rates for the patients over 70 years are shown in Table 4. Higher mortality rates compared to Boey *et al *reflect the more severely-ill group of patients in this later study.

#### b) Haceteppe score

The Haceteppe score for perforated peptic ulcers was developed from multi-variate analysis of 173 patients operated for perforated peptic ulcers, looking for variables associated with high mortality [16]. The four most-closely associated variables were the presence of a serious coexisting medical illness, acute renal failure, white cell count of more than 20 × 10^9^/l, and male sex. The score was established using these four variables. The sensitivity was 83%, the specificity 94%, and the overall predictive accuracy 93%. There has been no study to revalidate this score or test its accuracy against others.

### 9. Aneurysm scoring systems

#### a) Pre-operative POSSUM scores

The POSSUM score requires operative data to accurately predict outcome, but there are studies in vascular surgery on the pre-operative use of the score. In 2001 the Vascular Surgical Society of Great Britain and Ireland conducted an audit of 1345 vascular operations. One finding was that the physiological element of the POSSUM score predicted outcome of surgery as well as the total score with the operative element [35]. Neary *et al *showed that the POSSUM physiology score alone could predict the outcome of intra-arterial thrombolysis for acute leg ischaemia [36] and the same group in 2003 showed that the physiological POSSUM score predicted outcome for ruptured AAA surgery [37]. This suggests that pre-operative use of the physiological POSSUM score could be predictive, at least in emergency vascular surgery although none of these studies was exclusive to the elderly.

#### b) Hardman index

An Australian study from 1996 by Hardman *et al *[38] used logistic regression techniques on group of 154 patients with ruptured AAA, who were mostly over 65, to identify five independent pre-operative factors associated with mortality. These were age > 76, creatinine > 190 mmol/L, loss of consciousness, Haemoglobin < 9/dL and ECG signs of ischaemia; factors that can easily be collected before surgery. The presence of three or more risk factors was associated with 100% mortality. 0, 1 or 2 factors were associated with mortality risks of 16%, 37% and 72% respectively.

The Hardman Index has been compared with the physiological POSSUM score in predicting mortality after ruptured AAA surgery by Neary *et al *[36] who compared observed and predicted outcomes using each score on 191 cases of ruptured AAA undergoing emergency repair. They concluded that both scores predicted mortality well, but the Hardman Index was the easier to calculate at the bedside and would be easier to apply in practice, because it gives a clear prediction of death if three factors are present. However, in a recent study by Tambyraja *et al *[39] 9 patients with three or more criteria were operated on and 6 survived.

#### c) Glasgow Aneurysm Score (GAS)

First described in 1994, the GAS calculates a risk of mortality with ruptured AAA by using age in years + 17 for the presence of shock + 7 for myocardial disease + 10 for cerebro-vascular disease + 14 for renal disease [12]. Univariate analysis on 368 operated patients (ruptured and intact) was used to determine which risk factors carried greatest influence on outcome. The presence of shock was the single most important prognostic marker. By itself, rupture of the AAA was not a good predictor independently of shock. In a second study conducted prospectively, the same authors found that a score of 95 was correlated with a mortality of 80% [40].

A Finnish study of 836 patients with ruptured AAA showed that GAS accurately predicted mortality [41]. ROC curve analysis in this study showed that a score of 84 gave the greatest area under the curve for predicting death post-operatively. Mortality was 28% with a score of 84 or less; 65% with a score over 84 (p < 0.001). With a cut-off score of 85, Leo *et al *showed a mortality of 88.9% for ruptured AAA compared with 15.9% at 84 or less (p < 0.0001) [42].

GAS performed better than the Hardman Index in a study of 82 patients with a ruptured AAA undergoing surgery (median age 73), although neither was a good predictor of mortality [39]. It was also slightly superior in the study of 114 patients by Leo *et al *[42]. GAS is simple to apply to patients with ruptured AAA and could be applied prior to arrival in hospital. A score of 85 appears to be the best cut-off value between likely death and survival. Table 5 shows the observed mortality rates for each GAS score in the three largest studies to date.

**Table 5:** Comparison of observed post-operative mortality with GAS in the 3 largest studies

**GAS/Postoperative Mortality Rate by Age (years)**					
Korhonen^41^	n = 836	Samy^12^	n = 500	Samy^40^	n = 320
< 73	17%	< 70	6%	< 70	0%
74 – 82	35%	70 – 75	6%	70 – 75	2%
83 – 89	46%	76 – 85	16%	76 – 85	7%
90 – 97	61%	> 85	35%	86 – 95	36%
> 98	76%			> 95	80%

### 10. Cleveland clinic colorectal cancer model

Fazio *et al *used multivariate analysis of a database of 5034 patients (elective and emergency) to identify the six factors most associated with 30-day mortality in surgery for colorectal cancer [43]. Although two of these factors (TNM stage and whether or not the cancer is resected) may not be assessable pre-operatively, the authors describe their system as a pre-operative score and propose its use in emergency and elective surgery for colorectal cancer. Mortality risk can be calculated by measuring age, ASA, TNM stage, urgency of surgery, Haematocrit and resectability of tumour.

## Scores predicting morbidity

### 1. Veltkamp score

This model was developed on a cohort of 3075 patients of all ages, admitted to a general surgical ward for elective or emergency surgery. 11 patient, disease and surgery-related variables are used, with a reasonable predictive power for serious post-operative complications [44]. This score could be used pre-operatively as none of the 11 variables involve operative data. The model had an area under the ROC curve of 0.79. Minor complications were less-successfully predicted.

### 2. VA respiratory failure prediction index

The VA study was modelled on over 80, 000 men to devise a system to predict the 3.4% of patients who developed respiratory failure (defined as mechanical ventilation for 48 hours or more) after (non-cardiac) surgery [45]. Weighted scores are given for type of surgery, emergency surgery (less than 12 hours after admission), albumin, urea, pre-morbid functional status, respiratory function history and age. A score over 40 predicts a risk of respiratory failure of 31%. This score could be measured pre-operatively, but an estimate of the type of surgery would be required, although this would only need to decide on the location of surgery (thoracic, abdominal etc) rather than a specific procedure, with estimates of blood loss for example, as in the POSSUM score. The same authors have designed a pneumonia prediction score on a similar population of elderly male patients [46].

## Post-operative scores

Other scoring systems for attempting to quantify the risk of mortality or morbidity after emergency surgery have been described which are unsuitable for pre-operative assessment (Table 6). Only Cook and Day's scoring system [18] was developed in the context of an exclusively elderly group of emergency admissions.

**Table 6:** Variables needed to score four post-operative scoring systems

**Scoring system**	**Variables required**
Cook and Day^18^	Age, ASA, gender, ICU admission, invasive monitoring.
Mannheim Peritonitis Index ^28^	Age, gender, presence of organ failure/cancer, duration of pre-op peritonitis, origin of sepsis, nature of peritonitis/exudate.
Surgical Risk Score^47^	ASA, NCEPOD score, BUPA score

The Surgical Risk Scale (SRS; also called Surgical Risk Score by the same authors) proposed by Sutton *et al *[47] combines two elements that could be scored pre-operatively with one (the BUPA score of operative severity) that could be estimated pre-operatively if the likely operation was predictable. There is no recorded use of the SRS in this way, but as most emergency operations in elderly people would be classified as Major or Major-plus (BUPA 3/4) it may be possible to use the SRS hypothetically before surgery. Post-operatively the SRS had a similar accuracy to the P-POSSUM in the study by Brooks *et al *[48]. This score has not been studied in a cohort of exclusively elderly patients needing emergency operations.

## Discussion

There is no ideal scoring system for the pre-operative assessment of elderly patients needing emergency surgery. Some pre-operative scoring systems provide approximate estimates of mortality risk but none have been shown to be sufficiently specific for use on individual patients. At present, the Fitness Score has greatest specificity (80%) but would not be easy to use on all emergency admissions. Post-operative scoring systems such as P-POSSUM probably provide more accurate predictions, but are not useful in pre-operative assessment. Unfortunately, there are very few studies that have revisited old scoring systems or attempted to compare systems to assess which is best. Most articles in this field have proposed another new system.

The timing of data collection to create risk scores is seldom mentioned in the literature. Not only do physiological values vary during the acute admission, making the scores obtained by them unreliable, but there is evidence that to include operative findings and post-operative parameters on ICU improves the accuracy of the prediction. Although a score at initial assessment would help triage and plan treatment, comparative audit with post-operative scores remains the most useful function of scoring systems at present.

Even if accurate pre-operative predictions of outcome were possible by estimation of a risk score, an expert surgical opinion would be required to interpret these predictions at the bedside. An experienced clinician can not only assess prognosis but also weigh up the local facilities available, the patient's QOL and ethical issues, as well as considering the patient or relative's wishes. Scoring will never replace clinical judgement; if a prediction of 75% mortality after surgery is made by a score, it will still fall to the surgeon to decide whether or not to recommend an operation.

There is some evidence that expert surgical opinion is as accurate as any current pre-operative scoring system, or more so. Hartley and Sagar [49] compared surgical opinion on outcome after surgery, with a POSSUM score prediction. They showed that a surgeon's opinion had greater specificity than POSSUM at predicting death (88% vs 64%). Cook *et al *in an audit of mortality in the elderly tried to determine whether clinical judgement was better than scoring [50]. They found that pre-operatively, surgeons or anaesthetists predicted death with a specificity of 89%, which is greater than any of the scores identified in this review. Sensitivity was less good: 46% and 62% respectively. Markus *et al *found that surgeons tend to underestimate the risk of complications in emergency surgery, but that their clinical judgement was more accurate than P-POSSUM predictions [51] while Hobson *et al *studied 163 patients needing emergency surgery and compared predictions of 30-day mortality by surgeons, anaesthetists and POSSUM scoring and found that clinical predictions were as good as those made by scoring, using ROC curve analysis [52].

The specificity of surgical opinion will clearly depend on who the available surgeon is. If a senior surgeon is not available, then a scoring system may provide a better prediction. For that reason, scoring systems should continue to be developed.

Of the pre-operative scoring systems in use, the one which has stood the test of time and is used most, the ASA score, is also the most subjective, relying on the anaesthetist's overall clinical assessment of the patient. The fact that ASA scores vary between observers suggests that it is really an expert clinical assessment of risk and not a score at all. In a study of acutely perforated colorectal cancers in patients with a mean age of 70.5 years, stepwise logistic regression analysis showed that ASA scoring was the only significant pre-operative method of predicting short-term outcome [53].

Scoring systems such as the Reiss Index or Fitness Score can be used pre-operatively if there is time to gain enough data to complete the scoring. In future the speed and accuracy of investigations may allow a pre-operative diagnosis to be established more reliably, making these systems more useful than they are at present.

Scoring systems are generated and validated on specific populations that may be substantially different from the patients being scored in a different hospital. One potential resolution would be for each hospital to create a system specific to its own population, which is regularly re-validated.

One of the most accurate scoring systems used in elective cardio-thoracic surgery is the EUROSCORE [54]. It was developed from data on more than 19,000 operations in 128 centres. Because the surgery is elective and the variables most associated with mortality risk are clear, a high level of accuracy is possible and the score has been validated in North America and Europe [55]. Abdominal emergencies in the elderly are never going to be as predictable, and we must expect greater regional variation in this branch of surgery.

## Conclusion

There have been several attempts at creating a scoring system to predict mortality and morbidity risk after emergency surgery. Few of these have been specific to elderly patients. Some scoring systems provide a prediction that approximates to the observed mortality rate for a cohort, but none is sufficiently accurate to rely upon when considering an individual patient. In cases of ruptured AAA, the GAS comes closest to this ideal. Post-operative scoring has found its use in comparative audit but its advocates do not suggest its use on individual case prediction. Of the scores that can be applied pre-operatively, only the ASA is widely used. It provides the best balance of practicality and accuracy, especially if combined with another variable such as age or operative urgency. This may reflect the fact that the ASA score is primarily an anaesthetic opinion on the patient's overall state of health, and that clinical judgement is still critical to accurate risk prediction in the sick elderly patient.

## Abbreviations

AAA – Abdominal Aortic Aneurysm

ASA – American Society of Anaesthesiologists

APACHE – The Acute Physiology and Chronic Health Evaluation

BP – Blood Pressure

BUPA – British United Provident Association

CRI – Cardiac Risk Index

GAS – Glasgow Aneurysm Score

ICU – Intensive Care Unit

MPI – Mannheim Peritonitis Index

NCEPOD – National Confidential Enquiry into Operative Deaths

POSSUM – Physiological and Operative Severity Score for the enUmeration of Mortality

P-POSSUM – Portsmouth modification of POSSUM score

QOL – Quality of Life

ROC – Receiver Operator Characteristic

SA – Sickness Assessment

SAPS – Simplified Acute Physiology Score

SRS – Surgical Risk Scale/Score

TNM – Tumour Node Metastasis

## Competing interests

The author(s) declare that they have no competing interests.

## Authors' contributions

Both authors have made substantive contributions to the conception, writing and revising of this manuscript as well as giving final approval to the version submitted for publication.
